# Fertilization- and Irrigation-Modified Bacterial Community Composition and Stimulated Enzyme Activity of *Eucalyptus* Plantations Soil

**DOI:** 10.3390/ijms25031385

**Published:** 2024-01-23

**Authors:** Chunyu Huo, Jianhui Mao, Jianlang Zhang, Xinzhu Yang, Shangkun Gao, Jiyue Li, Qian He, Guangda Tang, Xianan Xie, Zujing Chen

**Affiliations:** Guangdong Key Laboratory for Innovative Development and Utilization of Forest Plant Germplasm, College of Forestry and Landscape Architecture, South China Agricultural University, Guangzhou 510642, China; 20223154029@stu.scau.edu.cn (C.H.); maojh@stu.scau.edu.cn (J.M.); zhangjl@stu.scau.edu.cn (J.Z.); yangxz@stu.scau.edu.cn (X.Y.); gaoshangkun@scau.edu.cn (S.G.); ljyue@scau.edu.cn (J.L.); heqian@scau.edu.cn (Q.H.); gdtang@scau.edu.cn (G.T.); 30004537@scau.edu.cn (X.X.)

**Keywords:** forest production, soil bacteria, diversity, correlation analysis, environmental factors

## Abstract

Irrigation and fertilization are essential management practices for increasing forest productivity. They also impact the soil ecosystem and the microbial population. In order to examine the soil bacterial community composition and structure in response to irrigation and fertilization in a *Eucalyptus* plantations, a total of 20 soil samples collected from Eucalyptus plantations were analyzed using high-throughput sequencing. Experimental treatments consisting of control (CK, no irrigation or fertilization), fertilization only (F), irrigation only (W), and irrigation and fertilization (WF). The results showed a positive correlation between soil enzyme activities (urease, cellulase, and chitinase) and fertilization treatments. These enzyme activities were also significantly correlated with the diversity of soil bacterial communities in *Eucalyptus* plantations.. Bacteria diversity was considerably increased under irrigation and fertilization (W, F, and WF) treatments when compared with the CK treatment. Additionally, the soil bacterial richness was increased in the *Eucalyptus* plantations soil under irrigation (W and WF) treatments. The Acidobacteria (38.92–47.9%), Proteobacteria (20.50–28.30%), and Chloroflexi (13.88–15.55%) were the predominant phyla found in the *Eucalyptus* plantations soil. Specifically, compared to the CK treatment, the relative abundance of Proteobacteria was considerably higher under the W, F, and WF treatments, while the relative abundance of Acidobacteria was considerably lower. The contents of total phosphorus, accessible potassium, and organic carbon in the soil were all positively associated with fertilization and irrigation treatments. Under the WF treatment, the abundance of bacteria associated with nitrogen and carbon metabolisms, enzyme activity, and soil nutrient contents showed an increase, indicating the positive impact of irrigation and fertilization on *Eucalyptus* plantations production. Collectively, these findings provide the scientific and managerial bases for improving the productivity of *Eucalyptus* plantations.

## 1. Introduction

Forest ecosystems are inhabited by a large number of soil microorganisms. Soil bacteria and fungi play essential roles in the cycling of materials and energy flow in forest ecosystems [1]. Microorganisms in the soil play crucial roles in decomposing organic materials, regulating mycorrhizal symbiosis, and facilitating the nitrogen cycle [2]. Bacteria contribute significantly to the conversion of plant biomass into organic matter through the decomposition of litter and soil [3]. This emphasizes the critical function that bacteria play in the transformation of organic materials in forests. Cellulolytic bacteria were successfully isolated from the topsoil of deciduous woodland, providing concrete evidence for their presence within common soil genera. These results show the significant function of these bacteria in breaking down cellulose in forest environments [4]. The most common phyla of soil microbes were found to be Proteobacteria and Acidobacteria [5]. In natural hardwood forests, the soil communities were dominated by Proteobacteria, while Acidobacteria dominated in coniferous forests [6]. The application of organic or compound fertilizers impacted the composition of bacterial communities in soil [7]. An investigation was conducted in three separate long-term fertilized sites within China’s Northeast Black Soil area. The findings revealed that the application of fertilizers not only led to a significant increase in microbial carbon biomass but also resulted in a higher proportion of symbiotic bacteria [8].

The intricate interplay between plants and soil microorganisms may have an enormous influence on the community structure, plant variety, and ecological function of an area [9]. In specific ecosystems, soil microbes and plants can undergo co-evolutionary processes [10]. Plants play a crucial role in providing nutritional substrates to soil microorganisms. The organic carbon and antimicrobial chemicals released by plant roots have been shown to directly influence the composition of bacterial communities in the rhizosphere [11]. Soil microbes break down over 90% of plant debris into inorganic nutrients essential for plant growth. This process not only increased a plant’s resistance to stress but also strengthens its defense against diseases [12,13]. Bacteria play a crucial role in the microbial communities associated with plants. They colonize both the endosphere and the root surface, known as the rhizosphere. Moreover, bacteria extensively spread throughout the rhizosphere, which is considered the primary site for microbial interactions in the soil immediately surrounding plant roots [14,15]. Research has shown that under drought stress, plant growth promoting rhizosphere bacteria can enhance plant drought tolerance [16]. Multi-omics analysis has led to the identification and characterization of key genes involved in plant–microbe interactions. This has deepened our understanding of how microorganisms adapt to their plant hosts [17,18].

Microorganisms in the soil serve as a sensitive predictor of both the productivity and condition of the soil [19]. Previous research suggested that microbial communities are influenced by soil fertility factors [20]. Soil enzyme activities are significant indicators of the functionality of the microbes in the soil as well as the fertility of the soil [21]. Some studies have shown that soil organic carbon (C) concentration positively correlated with the carbon-cycling enzymes and urease activity in an area that had received repeated applications of compost [22]. There has been a plethora of research on how soil microbes react following fertilization [23]. Replacing chemical fertilizers with organic fertilizers has been found to enhance microbial community interactions and potentially increase the functional diversity of soil ecosystems [24]. Fertilization affects soil microorganisms either indirectly through changes in soil characteristics or directly through the addition of nutrients [25]. The diversity in the soil’s bacterial community was shown to be associated with soil characteristics such as total carbon, nitrogen, phosphorus, potassium, and hydrogen (C, N, P, K, and pH) [26]. Researchers have discovered a strong link between the characteristics of the soil, like its overall nitrogen content, pH levels, and potassium availability, and the composition of bacterial communities in the soil [27]. Soil pH is a key factor influencing soil bacterial communities. Specifically, acidic soils have been found to have lower bacterial diversity compared to neutral and alkaline soils [28]. Acidobacteria are oligotrophic bacteria that exhibit a high abundance in environments with low nitrogen conditions [29]. Research has demonstrated that high levels of available phosphorous lead to lower soil bacterial diversity [30]. When compared to soil that has not been fertilized, organic fertilizer amendments tend to either preserve or increase the diversity in soil microbes [31]. By contrast, the application of chemical fertilizers is a “double-edged sword” and has both positive and negative effects on soil microbial diversity [32,33]. Water availability changes may influence soil microbial diversity, as soil extracellular enzyme and microbial activity can be reduced when soil moisture levels drop [34,35]. Meanwhile, water scarcity has detrimental effects on soil ecosystems, resulting in a reduction in organic carbon content and changes in the composition of microorganism communities [36].

*Eucalyptus* is used for large-scale afforestation in many countries due to its good dryness, fast growth, high yield, good resistance, etc. *Eucalyptus* is currently one of the top four fast-growing tree species globally used for afforestation. As of 2019, the total area of *Eucalyptus* plantations in China was over 5.4 × 10^6^ hectares [37]. Despite the abundant rainfall in southern China, there is still a seasonal dryness that significantly limits the output of *Eucalyptus* trees. The dry season is considered to be the primary factor influencing the vulnerability of *Eucalyptus* plantations [38]. *Eucalyptus* forest production was determined by management methods. Specifically, fertilization and irrigation were key management strategies to achieve high, stable, and sustainable yields of *Eucalyptus* plantations. Currently, *Eucalyptus* plantations have low productivity, and scientific and rational fertilization is one of the important measures for improving the efficiency of low-yield forest transformation. 

Soil management approaches have a direct influence on forest productivity through modified soil environmental characteristics and alter the composition and functions of microbial communities [39]. Consequently, it is essential to comprehend the changes in soil environment characteristics and bacterial composition in response to irrigation and fertilization in *Eucalyptus* plantations. This research is important because it will provide valuable insights to support the sustainable and green production of *Eucalyptus* plantations.

## 2. Results

### 2.1. Effects of Fertilization and Irrigation on Enzyme Activity in the Soil of Eucalyptus Plantations

The enzyme activities in the soil of a *Eucalyptus* plantations were measured under four different treatments of irrigation and fertilization (Table 1). The urease activity showed significant increases in the WF (25.20 ± 2.38 μg g^−1^ h^−1^) and F (18.72 ± 2.92 μg g^−1^ h^−1^) treatments compared to the CK (13.32 ± 1.12 μg g^−1^ h^−1^) and W (14.33 ± 1.31 μg g^−1^ h^−1^) treatments. The WF and F treatments exhibited urease activities that were 1.89 and 1.41 times higher than the CK treatment. Acid phosphatase activity was considerably diminished in the F treatment compared to the other treatments. Cellulase activity was significantly higher in the fertilization treatments (F and WF) compared to the non-fertilization treatments (CK and W). Chitinase activity showed a notable increase in the F, W, and WF treatments compared to the CK treatment, with the highest activity observed in the WF treatment (0.20 ± 0.02 μmol g^−1^ h^−1^), which was more than 2.22 times higher than the CK treatment. Invertase activity was significantly reduced in the F treatment compared to the CK treatment, while catalase activity increased significantly in the WF treatment. Peroxidase activity was notably lower in the F (4.53 ± 0.80 μmol kg^−1^ h^−1^) and W (5.94 ± 1.01 μmol·kg^−1^·h^−1^) treatments compared to the CK (7.55 ± 1.00 μmol kg^−1^ h^−1^) treatment.

### 2.2. Effects of Fertilization and Irrigation on the Bacterial Diversity in the Soil of the Eucalyptus Plantations

**Figure 1:**
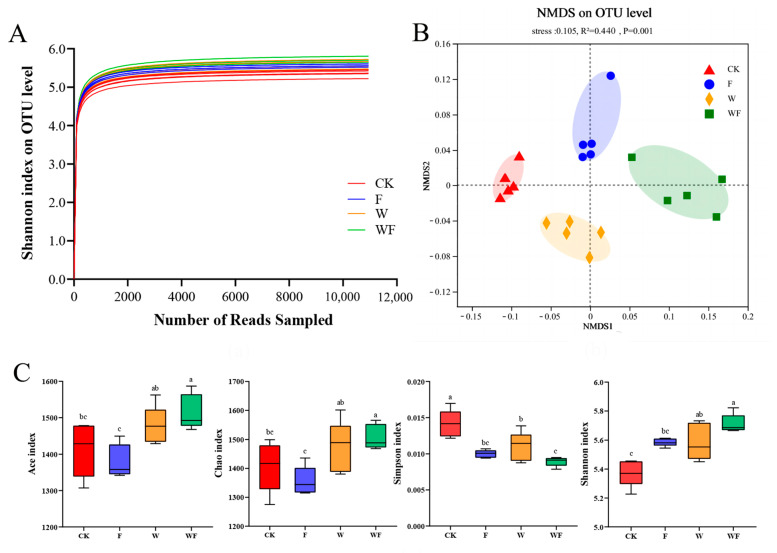
Rarefaction curves (**A**), non-metric dimensional scaling (NMDS) visualization of the β-diversity (**B**) and α-diversity analysis (**C**) of *Eucalyptus* plantations soil bacteria. The confidence interval for confidence ellipses is set at 95 percent. CK: control (no irrigation or fertilization); W: irrigation only; F: fertilizer only; WF: irrigation and fertilization. Results are shown as the mean ± standard deviation. In the box plots, the five horizontal lines represent the upper limit, 75th percentile, median, 25th percentile, and lower limit respectively, from top to bottom. The four treatments were compared for significance using the Duncan test, and different letters were assigned to indicate these significant differences in individual parameters (*p* < 0.05).

The rarefaction curves of 20 soil samples taken from the *Eucalyptus* plantations exhibited a gradual flattening, indicating sufficient sequencing depth for subsequent analyses (Figure 1A). According to the Analysis of β-diversity (NMDS), a clear separation was formed between each treatment (R^2^ = 0.440, *p* = 0.001), indicating significant differences in the bacterial population composition among the four treatments considered (Figure 1B). The WF treatment had the highest levels of Ace and Chao indicators, followed by the W treatment, as determined by operational taxonomic units (OTUs). The F, W, and WF treatments showed much lower Simpson index values compared to the CK treatment, while the Shannon index was considerably higher (Figure 1C).

### 2.3. Effects of Fertilization and Irrigation on Bacterial Community Composition in the Soil of the Eucalyptus Plantations

**Figure 2:**
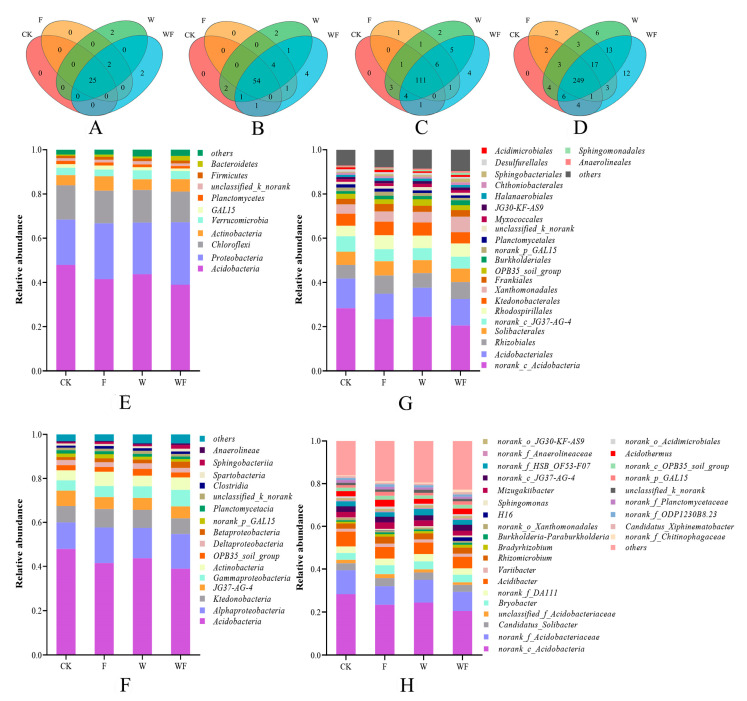
Venn diagram of soil bacteria of *Eucalyptus* plantations at the level of phylum (**A**), class (**B**), order (**C**), and genus (**D**) and relative abundance of the soil bacteria of *Eucalyptus* plantations at the level of phylum (**E**), class (**F**), order (**G**), and genus (**H**). CK: control (no irrigation or fertilization); W: irrigation only; F: fertilizer only; WF: irrigation and fertilization.

A total of 710,561 high-quality sequences were obtained in 20 soil samples collected from the *Eucalyptus* plantations under four treatments. These sequences were classified into 1879 operational taxonomic (OTUs). There were 25, 54, 111, and 249 OTUs overlapped in four treatments, accounting for 80.65%, 77.14%, 79.29%, and 76.62% of the total OTUs at the phylum, class, order, and genus levels, respectively (Figure 2A–D). Specifically, at the class level, there were two OTUs specific to the W and four OTUs specific to the WF treatments, representing 2.86% and 5.71% of the total OTUs, respectively (Figure 2B). At the order level, the F, W, and WF treatments had 1, 2, and 4 specific OTUs, respectively (Figure 2C). At the genus level, the F, W, and WF treatments had 2, 6, and 12 specific OTUs, accounting for 0.62%, 1.85%, and 3.7% of the total OTUs at that level, respectively (Figure 2D).

At the phylum level, Acidobacteria (38.92–47.9%), Proteobacteria (20.50–28.30%), and Chloroflexi (13.88–15.55%) dominated in the *Eucalyptus* plantations soil, accounting for over 73.30–91.75% in all bacterial phylum (Figure 2E). Compared with the CK treatment, the abundance ratio of Acidobacteria was significantly lower, while the proportion of Proteobacteria was significantly higher under fertilization and irrigation (F, W, and WF) treatments (*p* < 0.05) (Figure S1A). 

At the class level, Acidobacteria, Alphaproteobacteria, and Ktedonobacteria were found to be the dominant taxa in the soil of the *Eucalyptus* plantations (Figure 2F). In comparison to the CK treatment, the proportion of Acidobacteria tended to follow the order CK > W > F > WF, while the proportion of Alphaproteobacteria significantly increased under the fertilization treatments (F and WF) (*p* < 0.05) (Figure S1B). 

At the order level, the dominant orders were Acidobacteria (20.48–28.32%), Acidobacteriales (11.5–13.42%), and Rhizobiales (6.19–8.29%) (Figure 2G). Compared to the CK treatment, the proportion of Acidobacteria was significantly decreased under the fertilization and irrigation treatments (F, W, and WF). Conversely, the proportion of Rhizobiales was notably higher in the fertilization treatments (F and WF), while the proportion of Acidobacteriales was significantly lower in the F treatment (*p* < 0.05) (Figure S1C). 

At the genus level, the dominant genera observed in the soil of the *Eucalyptus* plantations were *norank_c_Acidobacteria* (20.48–28.32%), *norank_f_Acidobacteriaceae* (8.67–11.08%), and *Acidibacter* (5.41–7%) (Figure 2H). Compared to the CK treatment, the proportion of *norank_c_Acidobacteria* was significantly lower in the W, F, and WF treatments, while the proportion of *norank_f_Acidobacteriaceae* was notably lower in the F and WF treatments (*p* < 0.05) (Figure S1D).

**Figure 3:**
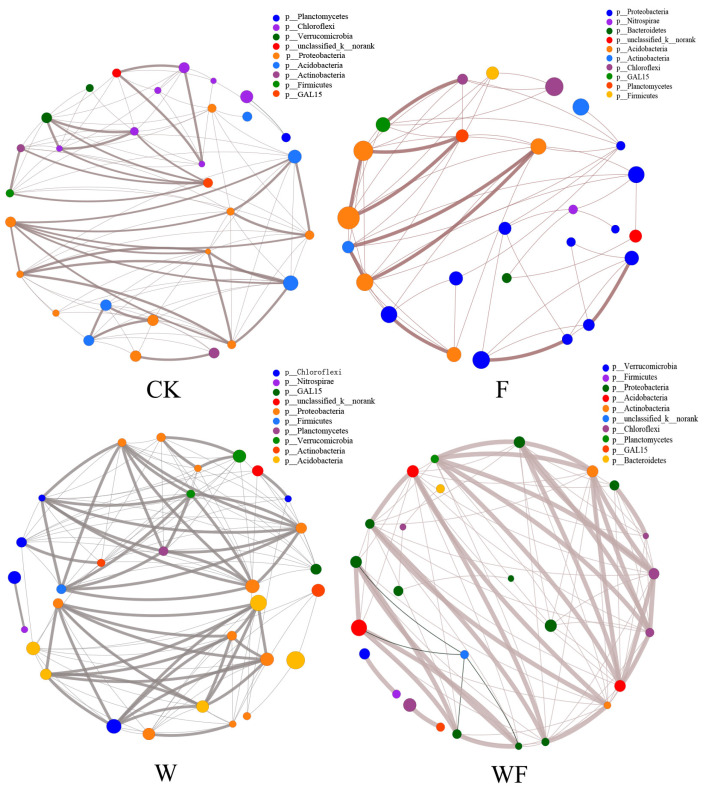
Correlation network displaying the relationship between the top 30 species in terms of their total abundance at the genus taxonomic level in the soil of the *Eucalyptus* plantations. The size of each node in the graph represents the abundance of the species, while different colors represent different phyla. Positive correlations are indicated by grey lines, while negative correlations are indicated by black lines. Thicker lines suggest a stronger link between species. Additionally, the number of lines connecting a species indicates its level of interconnectedness with other species. CK: control (no irrigation or fertilization); W: irrigation only; F: fertilizer only; WF: irrigation and fertilization.

The correlations of soil bacterial species among the four treatments in the soil of the *Eucalyptus* plantations also showed that Acidobacteria, Proteobacteria, and Chloroflexi were the dominant phyla in the four treatments. The correlation coefficients between the dominant phyla in the WF treatment were greater, and the bacterial species under the W treatment were more closely related (Figure 3).

### 2.4. Effect of Physicochemical Factors and Enzyme Activities on the Bacterial Community Structure in the Soil of the Eucalyptus Plantations

**Figure 4:**
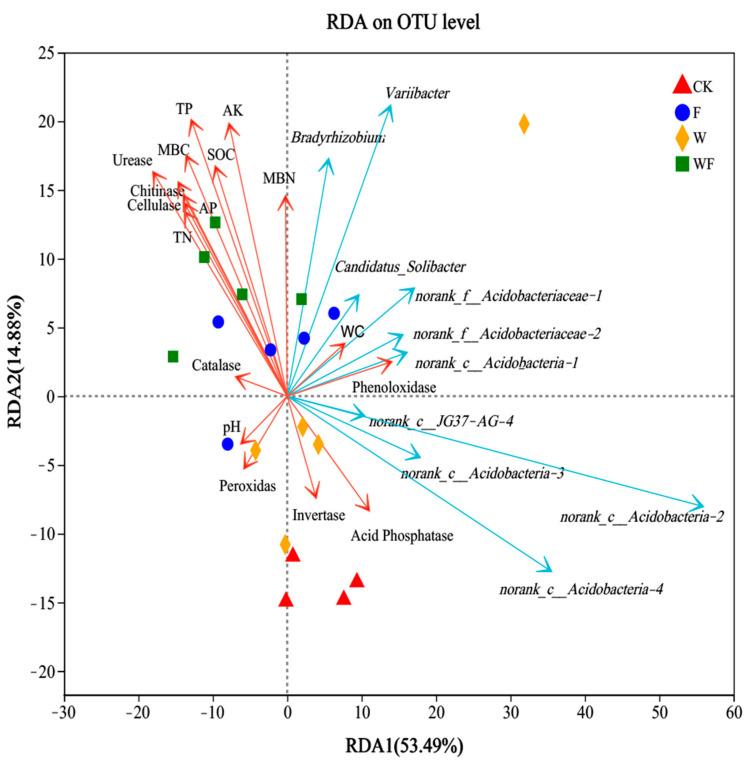
The redundancy analysis between soil environmental factors and bacterial community composition with the top ten dominant genera in *Eucalyptus* plantations soil under irrigation and fertilization. TN: total nitrogen; TP: total phosphorus; AP: available phosphorus; AK: available potassium; MBN: microbial biomass nitrogen; MBC: microbial biomass carbon; SOC: soil organic carbon; WC: field water capacity. CK: control (no irrigation or fertilization); W: irrigation only; F: fertilizer only; WF: irrigation and fertilization.

The soil physicochemical factors and enzyme activities significantly explained 68.37% of all variation in bacterial communities (Figure 4). The changes in the composition of bacteria communities along the first and second axes explained 53.49% and 14.88% of the total variation, respectively. The bacterial community in the soil of the *Eucalyptus* plantations was influenced by urease activity (*R*^2^ = 0.777, *p* = 0.001), TP (*R*^2^ = 0.76, *p* = 0.001), MBC (*R*^2^ = 0.65, *p* = 0.001), AK (*R*^2^ = 0.602, *p* = 0.001), chitinase activity (*R*^2^ = 0.601, *p* = 0.001), cellulase activity (*R*^2^ = 0.536, *p* = 0.001), AP (*R*^2^ = 0.506, *p* = 0.002), SOC (*R*^2^ = 0.493, *p* = 0.002), and TN (*R*^2^ = 0.484, *p* = 0.005) (Table S1). Urease activity, TP, MBC, AK, chitinase activity, cellulase activity, and the content of AP, SOC, and TN were favorably associated with the fertilization (F and WF) treatments. 

At the bacterial genus level, *Variibacter*, *Bradyrhizobium*, *norank_f_Acidobacteriaceae*, *Candidatus_Solibacter*, *norank_c_Acidobacteria*, and *norank_c_JG37-AG-4* were the dominant genera in the soil of the *Eucalyptus* plantations (Figure 4). *Variibacter, Bradyrhizobium*, *norank_f__Acidobacteriaceae*, *Candidatus_Solibacter*, and *norank_c_Acidobacteria* were positively correlated with the fertilization (F and WF) treatments, belonging to the phyla of Proteobacteria and Acidobacteria. *Norank_c_JG37-AG-4* and *norank_c_Acidobacteria* were positively correlated with the irrigation (W) treatment, belonging to the phyla of Chloroflexi and Acidobacteria. 

### 2.5. Correlation Analysis among Management Practices–Soil Factors–Bacterial Diversity

**Figure 5:**
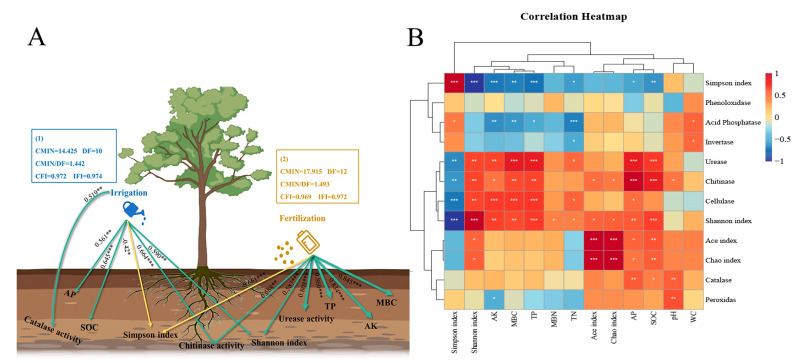
The effect of irrigation (1) and fertilization (2) on soil environmental factors and α-diversity of the soil bacterial (**A**) according to the structural equation model (SEM). Correlation analysis between the environmental factors and soil bacterial α-diversity of the *Eucalyptus* plantations under different treatments (**B**). The magnitude of the correlation is represented by the line’s thickness. The numbers adjacent to the arrows represent standardization regression weights. Positive and negative impacts are shown by the green solid and yellow dotted lines, respectively. ** *p* < 0.01, * *p* < 0.05, and *** *p* < 0.001 were used to denote the significant levels. TN: total nitrogen; TP: total phosphorus; AP: available phosphorus; AK: available potassium; MBN: microbial biomass nitrogen; MBC: microbial biomass carbon; SOC: soil organic carbon; WC: field water capacity.

The structural equation model (SEM) showed that irrigation had a significant impact on the contents of AP, SOC, and enzyme activities of chitinase and catalase in the soil of *Eucalyptus* plantations. The standardized regression weights for these variables were 0.561, 0.645, 0.664, and 0.519, respectively (Figure 5A). Furthermore, fertilization application, led to significant changes in the contents of TP, MBC, and AK, as well as the enzyme activities of urease and chitinase, with standardized regression weights of 0.860, 0.845, 0.834, 0.808, and 0.588, respectively, compared to the control (CK) treatment. Both irrigation and fertilization had a significant affect on the *Eucalyptus* plantations soil bacterial α-diversity. The contents of AP, MBC, TP, AK, and SOC showed significant positive correlations with urease and chitinase enzyme activity, respectively. Additionally, soil AP, MBC, TP, AK, SOC, and enzyme activities were positively correlated with the Shannon index but negatively correlated with the Simpson index (Figure 5B). 

### 2.6. Functional Analysis in the Soil of the Eucalyptus Plantations

According to the secondary taxonomic level of the KEGG database, there were 19 metabolic pathways with a relative abundance of metabolic-pathway-related genes greater than 1% in the *Eucalyptus* plantations soil (Figure S2). The top metabolic pathways in relative abundance were global and overview maps (40.11–40.96%), carbohydrate metabolism (9.25–9.41%), amino acid metabolism (7.56–7.69%), energy metabolism (4.31–4.39%), and the metabolism of cofactors and vitamins (4.21–4.26%). There were no significant differences in the metabolic pathways among the different treatments. The highest proportion of the global and overview maps consist mainly of metabolic pathways, biosynthesis of secondary metabolites, microbial metabolism in diverse environments, biosynthesis of amino acids, and carbon metabolism.

## 3. Discussion

### 3.1. Effect of Irrigation and Fertilization on the Enzyme Activity in the Soil of Forests

Soil enzyme activity serves as a barometer for different biochemical activities taking place in the soil. It is affected by elements such as the physical and chemical properties of the soil, its fertility status, and agricultural methods [40]. This study showed that fertilization management significantly enhanced the enzymatic activity of urease, cellulase, and chitinase in *Eucalyptus* plantations soil. (Table 1). The activity of the three enzymes showed a strong correlation with the composition of the bacterial population, particularly the Acidobacteria and Proteobacteria (Figure 4). Fertilization was linked to the activity of urease and chitinase enzymes, whereas irrigation exhibited a positive correlation with catalase and urease enzyme activities (Figure 5A). The application of irrigation and fertilization treatments has been shown to enhance soil nutrient content and increase microbial abundance. These changes in soil properties may be responsible for the observed increases in soil enzyme activity [41,42]. There was a proportional relationship between the quantity of fertilizer and irrigation and soil enzyme activity. Reasonable irrigation and fertilization could increase soil urease activity [42]. One study found that soil urease activity increased with a reduction in soil water content when fertilizer levels were low. However, under high fertilizer conditions, the highest soil urease activity was observed at 70–80% relative soil water content [43]. These results showed that fertilization and irrigation management strategies can stimulate enzyme activity and increase bacterial involvement in nutrient cycling and ecosystem function in *Eucalyptus* plantations. Bacteria are the primary mediators of chitin and cellulase degradation. Fertilization increased the soil bacterial community diversity, which may be responsible for the rise in soil chitinase and cellulase activities caused by fertilization [44]. The release of catalase enzyme is triggered by elevated levels of reactive oxygen species to protect against damage to DNA, proteins, and lipids [45]. High catalase activity is commonly regarded as a response to oxidative stress. The increase in soil water content caused by irrigation stimulated oxidative reactions in the soil, which might account for the increase in soil catalase activity under irrigation, but further research is need to test this hypothesis. This research demonstrated a strong positive connection among irrigation, fertilization, and soil physicochemical parameters (TP, AP, AK, MBC, and SOC); meanwhile, soil physicochemical properties (AP, MBC, TP, AK, and TN) were significantly positive correlated with soil enzymes activities (urease, cellulase, and chitinase). Collectively, these findings indicate that fertilization and irrigation might improve soil nutrient availabilities by increasing soil nutrient content and overall enzyme activity. These factors may be the potential reasons why irrigation and fertilization increase the productivity of *Eucalyptus* forests.

### 3.2. Effect of Irrigation and Fertilization on Soil Bacterial Community Composition

Soil type is a particularly important element in determining soil microbial diversity [46] as well as the fertilization program [47] and irrigation technique [48]. Microbial taxonomic and functional diversity have the potential to influence soil and ecosystem processes. The results revealed that fertilization and irrigation greatly increased bacterial diversity in the soil; meanwhile, irrigation specifically increased the richness of soil bacteria (Figure 1C). Previous research has demonstrated that changes in soil moisture levels can directly and indirectly influence bacterial abundance and community composition [49]. Specifically, irrigation has been found to enhance nutrient availability, create a fluctuation between dry and wet soil conditions, and promote the proliferation of soil bacteria [50]. The RDA findings of our research revealed that several soil variables may impact the community of bacteria composition (Figure 4). Soil physicochemical characteristics substantially influenced the bacterial community structure [51] and fertilization raised the soil’s nutrient content, which enhanced the bacterial diversity.

At the phylum level, the predominant bacteria observed in this study were Acidobacteria, Proteobacteria, and Chloroflexi. Interestingly, when compared to the CK treatment, both fertilization and irrigation resulted in a significant decrease in the proportion of Acidobacteria and a substantial rise in the proportion of Proteobacteria (Figure 2E and Figure S1). Acidobacteria and Proteobacteria were the most numerous soil bacterial groupings and the main crop-associated rhizosphere bacteria [52]. According to our research, the most common kind of *Eucalyptus* plantations soil bacteria was Acidobacteria, which accounted for the highest proportion. Acidobacteria, classified as oligotrophic bacteria, are typically found in nutrient-poor and highly acidic soil environments. They have the ability to degrade complex and resistant carbon sources, thereby facilitating the decomposition of organic matter in the soil [53]. Acidobacteria have been identified in various environments and constitute approximately 20–50% of the bacterial community in soil. Their presence plays a crucial role in the development and maintenance of soil ecosystems [54]. Previous studies have shown a positive correlation between Acidobacteria and soil pH [55]. In this study, the proportion of Acidobacteria phylum was markedly reduced after F and WF treatment. This decrease can be attributed to the fact that fertilizer application has the potential to lower soil pH [56]. In this investigation, it was found that irrigation had a significant impact on reducing the relative abundance of Acidobacteria. This finding is consistent with previous research, which also observed a decrease in Acidobacteria proportions despite an increase in soil water content [57]. The study revealed that irrigation resulted in the stimulation of net primary productivity. This increase in productivity led to higher availability of carbon in the soil, which in turn favored the growth of copiotrophic microorganisms over oligotrophic ones [58]. In a semi-arid mountain pine forest, long-term watering was found to reduce the proportions of Acidobacteria [59]. Irrigation has been shown to enhance soil carbon mineralization. This could be attributed to the increase in Proteobacteria and decrease in Acidobacteria proportions observed with irrigation.

Proteobacteria were prevalent in nutrient-rich settings [59]. Proteobacteria exhibit a rapid reactivity to unstable carbon and phosphorus resources [60], enabling them to grow rapidly and adapt to a wide range of soil conditions. Several prokaryotic bacteria have been demonstrated to be beneficial for plant development in previous research. Bacilli, a beneficial microorganism, plays a vital role in plant development and reduces the occurrence of plant diseases through a variety of processes [61]. In soils, Proteobacteria are essential to the nitrogen (N) cycle as they included both autotrophic and heterotrophic ammonium-consumers [62]. In the present study, the population of the Proteobacteria considerably increased with both fertilization and irrigation (Figure 2A and Figure S1). Fertilization practices have a significant impact on the nitrogen cycle of soil. Both inorganic and organic fertilizers facilitated processes such as nitrogen fixation and anaerobic ammonia oxidation [63]. Numerous studies have shown a positive correlation between the use of chemical fertilizers and the proliferation of Proteobacteria. This suggests that chemical fertilizers enhance soil carbon storage and nitrogen cycling, as well as promote the production of polysaccharides and microbial slime that help maintain soil aggregates [64]. Meanwhile, several studies have demonstrated that irrigation increased the proportions of Proteobacteria [65]. In this study, there was a positive correlation between soil physicochemical properties and Proteobacteria (Figure 4). This finding is consistent with previous studies that have also reported a positive correlation between Proteobacteria and soil nutrient availability, such as total carbon (TC) and total nitrogen (TN) [66,67]. This work demonstrated that irrigation and fertilization changed the bacterial community in *Eucalyptus* plantations soil. We hypothesized that an increase in carbon availability in the soil under irrigation conditions may have led to changes in the abundance of Acidobacteria and Proteobacteria. The decrease in the proportions of Acidobacteria under fertilization conditions may be attributed to changes in soil pH. The increase in the relative abundance of Proteobacteria under fertilization conditions may be associated with the increased soil carbon content and nitrogen cycling. 

### 3.3. Effect of Irrigation and Fertilization on the Soil Bacterial Function

We used KEGG functional gene annotation to predict the functional gene in soil bacteria under irrigation, fertilization, and CK treatment (Figure S2). The results showed no significant differences in functional genes between the treatments, which may be related to gene redundancy [68]. Although there may be significant changes in the species composition of soil microbial communities in response to environmental changes, the functional genes within these communities remain relatively stable. The analysis of the KEGG dataset revealed that the predominant metabolic pathways in soil microbial communities were carbohydrate metabolism, amino acid metabolism, energy metabolism, and vitamin and co-factor metabolism. Additionally, membrane transport and signal transduction pathways were identified as key components involved in the processing of environmental information [69]. The experimental results revealed that the main metabolic pathways in the soil of the *Eucalyptus* plantations include carbohydrate metabolism, amino acid metabolism, energy metabolism, and the metabolism of cofactors and vitamins. These pathways accounted for a significant proportion of the total metabolic pathways, as shown by the global and overview maps. Additionally, the metabolic pathways identified in this study encompassed the biosynthesis of secondary metabolites, microbial metabolism in diverse environments, biosynthesis of amino acids, and carbon metabolism. By examining the genetic level and predicting related metabolic pathways, this study offers an initial understanding of the meso-energetic metabolic processes of bacteria in *Eucalyptus* plantations soil. The findings of this study can serve as fundamental data and references for future research on soil species composition and material cycling.

## 4. Materials and Methods

### 4.1. Study Site and Experimental Materials

The experimental site is located within the teaching and research base of South China Agricultural University, which is situated in the Zengcheng District of Guangzhou (23°14′48″ N, 113°38′20″ E). The study area has a subtropical monsoon climate, with an average annual rainfall of 2004.5 mm and an annual average temperature of 21.91 degrees Celsius [70]. The area experienced a prominent rainy season from April to September, while the dry season occurs between October and March. During the dry season, which accounts for approximately 17.31% of the total annual rainfall, the region faces severe water shortage and seasonal drought. 

The experimental plant material used was a three-month-old clone (DH32-29) of *Eucalyptus urophylla × Eucalyptus grandis*. The study area spanned 5360 square meters and had typical red loam soil. Soil samples were collected from a depth of 0 and 20 cm below the surface. The main soil properties were pH 4.92, 7.03 g kg^−1^ organic matter, 0.35 g kg^−1^ total nitrogen, 0.15 g kg^−1^ total phosphorus, 8.83 g kg^−1^ total potassium, 204.1 g kg^−1^ field water holding capacity, and 1.55 g cm^−3^ soil volumetric weight. In summary, the study area had a slightly acidic pH and high levels of organic matter and potassium. However, it was deficient in nitrogen and phosphorus. The location, climate, and soil properties of the area were essential considerations for this study.

### 4.2. Experimental Method 

A detailed description of the orthogonal experimental design for irrigation and fertilization in this experiment can be found in the work conducted by Yu et al. [70]. The horizontal terrace preparation method was described by Hua et al. [71]. The experiment consisted of five terraces, with each terrace divided into four treatments (CK, W, F, and WF). For treatments F and WF, the base fertilizer was applied in March 2017, with a composition of 24 g of nitrogen (N), 72 g of phosphorus (P), and 24 g of potassium (K). Topdressing was applied in July 2017 with the same composition of effective elements. Each plant received a base fertilizer application of 400 g and a top-dressing application of 300 g. During the experiment, drip irrigation was used between October 2017 and January 2018, which represented the dry season. The drip irrigation rate was set at 4 L per hour for a total of 8 h per week, resulting in a total weekly irrigation volume of 32 L. In total, each plant received a total of 512 L of water throughout the experiment. 

On 7 January 2018, soil sampling was conducted in the *Eucalyptus* plantations. Each treatment had 5 replicates, resulting in a total of 20 plots. With each plot, 8 sampling points were randomly selected. The dead soil covering on the soil surface was removed, and soil samples were collected from the top 0–20 cm layer. The soil samples collected from each treatment plot were thoroughly mixed to ensure homogeneity. Any debris such as gravel, tree roots, and dead leaves were removed, and the samples were stored in bags. The soil samples obtained from each plot were partitioned into two portions. One portion of the samples was cryogenically frozen in liquid nitrogen and promptly refrigerated at −80 °C upon its return to the laboratory for analysis of microbial diversity and soil DNA extraction. The remaining samples were transported to the laboratory, where the soil moisture content was promptly assessed. The remaining samples were then dried and preserved for future examination of soil physical and chemical characteristics, enzyme activity, and soil microbial biomass.

### 4.3. Soil Enzyme Activity and Physicochemical Properties 

Enzyme activity refers to the capacity of an enzyme to catalyze specific chemical reactions. It is commonly measured by determining the rate of enzymatic conversion, which can be expressed as the decrease in substrate or the increase in product per unit volume within a given time period. Soil urease activity was determined using the sodium phenol–sodium hypochlorite colorimetric method [72], and expressed as NH_4_^+^ production per gram of dry soil per unit time (μg g^−1^ h^−1^). The sodium p-nitrophenol phosphate method was used to measure soil acid phosphatase activity [73], with enzyme activity expressed as p-nitrophenol produced per unit time per gram of dry soil (μmol g^−1^ h^−1^). Soil cellulase and invertase activities were determined by the 3,5-dinitrosalicylic acid colorimetric method [74]. Chitinase and catalase activities were determined by potassium permanganate titration [75]. The colorimetric method was utilized to assess and measure the activity of phenoloxidase [76], and peroxidase activity was determined by the guaiacol method [77]; these were expressed as dopachrome production per kilogram of dry soil per unit time (μmol·kg^−1^·h^−1^). The determination of these soil physicochemical properties followed the methods described in previous studies [56]. The evaluation of soil total nitrogen (TN) and soil organic carbon (SOC) followed the methodology outlined by Finzi et al. [78]. The NaOH melting-molybdenum antimony colorimetric method was employed to analyze and quantify the levels of TP and TK in the samples. The ascorbic acid reductant method was employed to extract and determine the concentration of AP, while an atomic absorption spectrophotometer was utilized to measure the concentration of AK [79]. Techniques from previous studies were employed to determine the levels of MBN and MBC [80]. Each treatment for soil enzyme activity and physicochemical property determination was replicated five times.

### 4.4. Soil DNA Extraction and ILLUMINA Sequencing

To extract the total DNA from the soil samples, we utilized the Power Soil DNA Isolation Kit (MoBio Laboratories, Carlsbad, CA, USA). Each sample consisted of approximately 0.5 g of soil. The quality and concentration of the DNA were assessed using a Nanodrop 2000 Spectrophotometer (Nanodrop Technologies, Wilmington, DE, USA). Moreover, agarose gel electrophoresis was conducted on 10 g L^−1^ gels to evaluate the purity and integrity of the DNA. The DNA samples were appropriately diluted with sterile water to achieve a final concentration of 1 nanogram per microliter (1 ng/μL). For the amplification of the V3-V4 region of the 16S rRNA gene in bacteria, the primers 338F and 806R were used [7]. The PCR amplification was performed using TransGen AP221-02: TransStart Fastpfu DNA Polymerase, with a 20 μl reaction system. The PCR was conducted using the ABI GeneAmp® 9700 PCR machine. The PCR reaction parameters were as follows: 1× (3 min at 95 °C); 27 × (30 s at 95 °C; 30 s at 55 °C; 45 s at 72 °C); 10 min at 72 °C, then cooled to 10 °C until halted by the user. Standard experimental procedures were followed for all samples, with three replicates performed for each sample [81]. 

After pooling the PCR results from the same sample, analysis was conducted using 2% agarose gel electrophoresis. The desired PCR products were extracted from the gel using the AxyPrep DNA Gel Recovery Kit (AXYGEN), followed by elution with Tris-HCl buffer. Finally, the eluted DNA was subjected to 2% agarose gel electrophoresis for further analysis. The three amplicon libraries, with equimolar concentrations, were combined into a single mixture. Subsequently, sequencing of the mixture was performed on the Illumina MiSeq platform using 2 × 300 nt paired-end reads (Illumina, San Diego, CA, USA) at Shanghai Majorbio Bio-Pharm Technology Co. Ltd. The number of optimized sequences after quality control splicing of bipartite sequences was 710,561, the number of optimized sequence bases was 293,073,992, and the average length of the sequences was 412 bp.

### 4.5. Data Analysis

The raw sequencing data underwent sequence splicing and quality control using Flash (Fast Length Adjustment of Short) (https://ccb.jhu.edu/software/FLASH/index.shtml, accessed on 16 November 2017). Uparse (http://www.drive5.com/uparse/, accessed on 29 November 2017) was employed for sequence analysis, grouping sequences with more than 97% similarity into operational taxonomic units (OTUs). To obtain the taxonomic information of each OTU, we utilized the RDP classifier (version 2.11 http://sourceforge.net/projects/rdp-classifier/, accessed on 3 January 2018) to perform taxonomic analysis on the representative sequences of OTUs with a 97% similarity level. We compared the community species composition of each sample at every taxonomic level using Silva (Release 138, http://www.arb-silva.de, accessed on 10 January 2018), RDP (Release 11.5, http://rdp.cme.msu.edu/, accessed on 10 January 2018), and Greengene (Release 13.5, http://greengenes.secondgenome.com/, accessed on 18 January 2018). Alpha diversity indices for the samples were calculated using the Mothur (1.30.2) software package, available for download from the Mothur website (https://www.mothur.org/wiki/Download_mothur, accessed on 25 January 2018). To assess the differences in bacterial community composition between treatments, the non-metric multidimensional scaling (NMDS) method were employed. Additionally, redundancy analysis (RDA) was conducted to evaluate the influence of environmental factors on the structure of the soil bacterial community by considering them as environmental variables. Species distribution and functional analysis were plotted using GraphPad Prism 8.0. (GraphPad Software, San Diego, CA, USA) Structural equation modeling paths were produced using Amos Graphics (IBM SPSS Amos 22.0.0) and correlation clustering heat map analysis was performed using the OmicStudio tools (version 3.6 https://www.omicstudio.cn/tool, accessed on 20 March 2023). The correlation network diagram was constructed using Spearman correlation analysis, while the correlation heatmap was generated using Pearson correlation analysis. For soil enzyme activity, microbial α-diversity, and dominant bacterial abundance, statistical analysis was conducted using the IBB SPSS program. One-way analysis of variance (ANOVA) and the Duncan test (*p* < 0.05) were performed, and different letters indicate differences between means. All data are shown with means and standard errors.

## 5. Conclusions

This study demonstrated that irrigation and fertilization influenced the enzyme activities and bacteria community structure in *Eucalyptus* plantations soil. Eenzyme activities of urease, cellulase, and chitinase were all markedly upregulated by the fertilization treatment. The chitinase and catalase enzyme activities were favorably associated with irrigation. The alterations in enzyme activity may be associated with enhancements in soil nitrogen and carbon levels due to fertilization; meanwhile, irrigation created a favorable wet environment that may have promoted nutrient absorption and expedited enzymatic reactions. Moreover, both irrigation and fertilization significantly increased soil bacterial diversity, and irrigation particularly impacted soil bacterial richness. Acidobacteria (oligotrophic), Proteobacteria (copiotrophic), and Chloroflexi were the dominant phyla in the *Eucalyptus* plantations soil. The decrease in Acidobacteria richness could be attributed to the increase in soil carbon content and changes in pH under irrigation and fertilization treatments. Conversely, the enrichment in Proteobacteria might be due to alterations in soil carbon storage and nitrogen cycling. Overall, the applications of fertilization and irrigation increased soil carbon and nutrient contents, stimulated overall soil enzyme activity, and modified the soil bacterial community, leading to a soil environment that promoting *Eucalyptus* growth.

By analyzing the effects of irrigation fertilization on soil enzyme activities and soil bacterial communities, this study aimed to clarify the community structure of soil bacteria in *Eucalyptus* plantations and identify the main environmental factors influencing them. The findings of this study offer valuable insights for the implementation of effective water and fertilizer management practices, thus contributing to the sustainable and environmentally friendly development of *Eucalyptus* plantations.

## Figures and Tables

**Table 1:** Enzyme activities in *Eucalyptus* plantations soil under fertilization and irrigation application.

Treatment	CK	F	W	WF
Urease (μg g^−1^ h^−1^)	13.32 ± 1.12 c	18.72 ± 2.92 b	14.33 ± 1.31 c	25.20 ± 2.38 a
Acid Phosphatase (μmol g^−1^ h^−1^)	5.77 ± 0.50 a	4.14 ± 0.65 b	5.83 ± 0.46 a	5.20 ± 0.19 a
Cellulase (μmol g^−1^ h^−1^)	0.07 ± 0.01 b	0.09 ± 0.01 a	0.08 ± 0.01 b	0.09 ± 0.01 a
Chitinase (μmol g^−1^ h^−1^)	0.09 ± 0.01 c	0.11 ± 0.01 b	0.11 ± 0.01 b	0.20 ± 0.02 a
Invertase (mg g^−1^ d^−1^)	4.88 ± 0.93 a	3.31 ± 0.30 b	5.39 ± 1.57 a	4.79 ± 0.38 a
Catalase (mg g^−1^ 20 min^−1^)	0.70 ± 0.04 b	0.69 ± 0.04 b	0.72 ± 0.02 ab	0.76 ± 0.04 a
Phenoloxidase (μmol kg^−1^ h^−1^)	4.07 ± 1.45 ab	4.43 ± 0.87 ab	5.41 ± 1.22 a	3.63 ± 1.06 b
Peroxidase (μmol kg^−1^ h^−1^)	7.55 ± 1.00 a	4.53 ± 0.80 b	5.94 ± 1.01 b	7.44 ± 1.41 a

CK: control (no irrigation or fertilization); W: irrigation only; F: fertilizer only; WF: irrigation and fertilization. Results are shown as the mean ± standard error. Using the Duncan test, different letters meant that the factors changed significantly among the four strategies (*p* < 0.05).

## Data Availability

Each sample’s raw sequence files can be found in the NCBI SRA BioProject database (Accession Number: PRJNA637913).

## Supplementary Materials

The following supporting information can be downloaded at: https://www.mdpi.com/article/10.3390/ijms25031385/s1.
