# Supplementary material for: Fertilization- and Irrigation-Modified Bacterial Community Composition and Stimulated Enzyme Activity of Eucalyptus Plantations Soil
Source: Int J Mol Sci. 2024 Jan 23;25(3):1385. doi: 10.3390/ijms25031385 (PMC10855151; doi:10.3390/ijms25031385)
Supplement: Supplementary file 1 [file ijms-25-01385-s001.zip › ijms-2826771-supplementary.pdf]

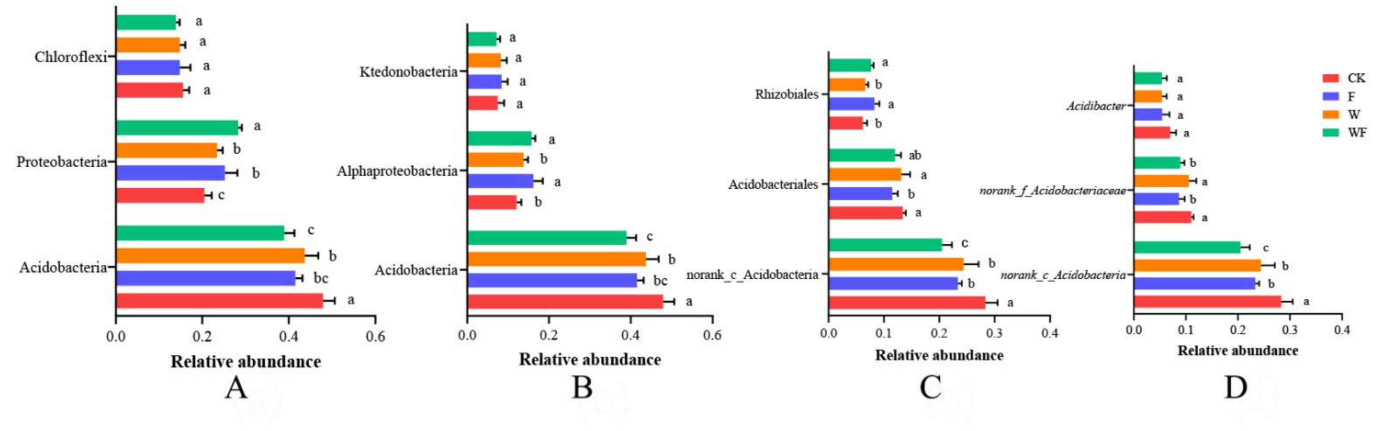

Figure S1. Top three relative abundances of soil bacteria at the level of phylum(A), genus(B), order (C), genus (D). Different letters indicate significant differences in individual parameters among the four treatments detected using the Duncan test ($P < 0.05$). The mean ± standard deviation was shown. CK: control (no irrigation or fertilization); W: irrigation only; F: fertilizer only; WF: irrigation and fertilization.

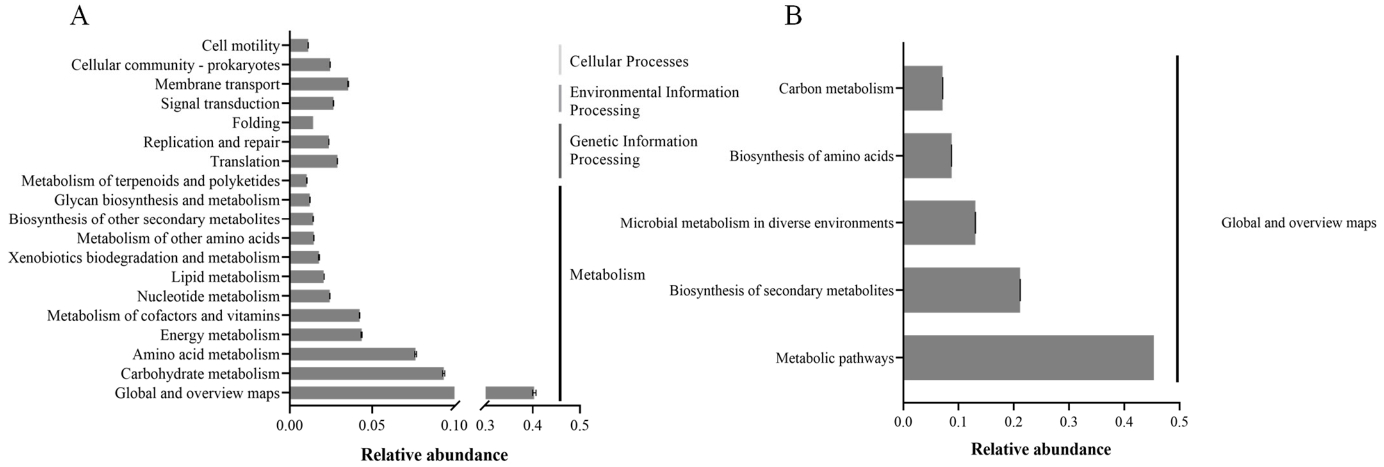

Figure S2. The distribution of bacterial metabolic pathways at the secondary taxonomic level was analyzed using the Kyoto Encyclopedia of Genes and Genomes (KEGG) (A); Top 5 metabolic pathways in the global and overview map (B).

Table S1. Correlation analysis between top ten dominant microbial species and environmental factors in the soil of *Eucalyptus* plantations

Environmental Factors	RDA1	RDA2	R^2	P-value
WC	0.899	0.437	0.081	0.534
pH	-0.874	-0.487	0.054	0.640
MBC	-0.600	0.800	0.650	0.001
MBN	-0.064	0.998	0.271	0.093
TN	-0.696	0.719	0.484	0.005
TP	-0.535	0.845	0.759	0.001
AP	-0.681	0.733	0.506	0.002
AK	-0.381	0.925	0.602	0.001
SOC	-0.500	0.866	0.493	0.002
Urease	-0.719	0.695	0.777	0.001
Acid Phosphatase	0.773	-0.634	0.242	0.116
Cellulase	-0.671	0.741	0.536	0.001
Chitinase	-0.667	0.745	0.601	0.001
Invertase	0.468	-0.884	0.088	0.464
Catalase	-0.967	0.256	0.055	0.632
Phenoloxidase	0.991	0.137	0.234	0.132
Peroxidas	-0.723	-0.691	0.068	0.565

* The $P-$alues of RDA1 and RDA2 were the cosine of the angle between the soil environmental factor arrow and the sorting axis; R^2 represented the determination coefficient of environmental factors on species distribution. WC: field water capacity; MBC: microbial biomass carbon; MBN: microbial biomass nitrogen; TN: total nitrogen; TP: total phosphorus; AP: available phosphorus; AK: available potassium; SOC: soil organic carbon.
